# LINC00152 Knock-down Suppresses Esophageal Cancer by EGFR Signaling Pathway

**DOI:** 10.1515/med-2020-0019

**Published:** 2020-03-06

**Authors:** Yan Ding, Hai Guo, Liangjun Zhu, Li Xu, Qingyu Pei, Youjun Cao

**Affiliations:** 1 Department of Oncology, BenQ Medical Center, The Affiliated BenQ Hospital of Nanjing Medical University, Nanjing 210019, China; 2 Nanjing University of Chinese Medicine, Nanjing 210046, Jiangsu Province, China; 3 Department of Internal Medicine, Huai’an First People’s Hospital, Huai’an 223300, Jiangsu Province, China; 4 Department of Internal Medicine, Jiangsu Provincal Tumor Hospital, Nanjing 210009, Jiangsu Province, China; 5 Department of Orthopaedics, Nanjing Yuhua Hospital, Nanjing 210000, Jiangsu Province, China

**Keywords:** lncRNA LINC00152, Esophageal cancer, Eca 109, Kyse 150, EGFR pathway

## Abstract

**Aim:**

This study aims to explain the role and mechanism of lncRNA LINC00152 in esophageal cancer.

**Methods:**

The 30 pairs of esophageal cancer and adjacent normal tissues were collected and measuring the lncRNA LINC00152 expression by ISH and RT-qPCR assay. In the next cell experiment, Eca 109 and Kyse 150 cells were divided into 3 groups: NC group were treated with non-treatment; BL group were transfected with empty vector and lncRNA group were transfected with lncRNA LINC00152. The cells proliferation were measured by MTT assay; the cells apoptosis and cell cycle were evaluated by flow cytometry. The relative proteins expressions were measured by WB assay.

**Results:**

Compared with NC groups, the cell proliferation rate of lncRNA groups were significantly suppressed (P<0.05, respectively); the cell apoptosis and G1 phase rates were significantly enhanced in the lncRNA groups (P<0.05, respectively). In the proteins expressions, the EGFR, PI3K and AKT proteins expressions of lncRNA group were significantly inhibited and the P21 proteins expressions were significantly stimulated in the lncRNA groups compared with those of NC groups in Eca 109 and Kyse 150 cells.

**Conclusion:**

The lncRNA LINC00152 had anti-tumor effects on esophageal cancer in the Eca 109 and Kyse 150 cells, the mechanisms were relative with EGFR pathway.

## 1 Introduction

Esophageal cancer is a common gastrointestinal malignancy [1], which accounts for more than 70% of esophageal cancer patients worldwide [2]. In recent years, many studies had confirmed that lncRNas were closely related to human tumors, and lncRNA had unique biological functions in cancer suppression and cancer promoting pathways. Therefore, lncRNAs had become a new hotspot in cancer research in recent years. Related studies reported that lncRNA is closely related to the occurrence, development, metastasis and recurrence of esophageal cancer [3, 4, 5]. Therefore, the study of the expression and its mechanism of lncRNA has become an important theoretical basis for molecular diagnosis and molecular targeted therapy of esophageal cancer. LncRNA LINC00152 plays an important role in lncRNAs family. Some relevant studies reported that the lncRNA LINC00152 was over-expression in some cancer tissues by regulation of relative pathways to promote cancer cells proliferation [6, 7, 8, 9]. In this study, we discussed the effects and mechanisms of lncRNA LINC00152 knockdown in Eca 109 and Kyse 150 cells which were two kinds of esophageal cancer in vitro study.

## 2 Materials and methods

### 2.1 Clinical sample

Thirty pairs of Non-tumor and esophageal cancer tissues were taken from esophageal cancer patients who received treatment in Nanjing University of Chinese Medicine from October 2015 to December 2016. All patients were primary esophageal carcinoma and un-treated before the operation. The tissues were resection and quickly stored in the 10% polyoxymethylene to fix until using.

### 2.2 In situ hybridization (ISH) assay

The tissues were embedded by paraffin and section as 5μm, sections dewaxing by xylene, after gradient alcohol hydration, using fresh 3% H_2_O_2_ to close and place at room temperature for 5-10 min; washing by distilled water (2 min×3 times); adding a drop fresh pepsin which was dilution by 30 g/l citric acid to incubate in the thermostat box for 10-30 min; ISH PBS was used to wash (5 min×3 times), washing by distilled water for 2 min; adding pre-hybrids as 20 μl/slice to place in the wet box to culture in the thermostat box (40°C) for 3h; adding pre-hybrids as 20 μl/slice, special cover film for in-situ hybridization and place in the wet box in thermostat box (40°C) overnight; Gently uncover the special cover film of the in situ hybridization (2×SSC buffer solution as 5 min×2 times; 0.5×SSC buffer solution for 15 min; 0.2×SSC buffer solution for 15 min); adding closed liquid as 30μl/slice to culture for 30 min at 37°C; getting rid of the excess liquid, adding 30μl/slice biotinylated rat digoxin to culture for 1 h at 37°C; ISH PBS was used to wash (5 min×4 times); adding SABC 30μl/slice to culture for 30 min at 37°C; washing by ISH PBS (5 min×4 times); adding fresh DAB to color, washing by water; Gradient alcohol dehydration, Xylene transparent, Neutral gum seal. Using image analyze software to analysis the IOD of different tissues.

### 2.3 RT-qPCR assay

Total RNA was extracted from clinical samples using Trizol reagent (Thermo Fisher Scientific) following the protocols of manufacturer. For the measurement of linc00152 mRNA level, RNA was reversely transcribed into cDNA using M-MLV Reverse Transcriptase (Thermo Fisher Scientific) together with random primers and then cDNA was used for qPCR detection by PowerUp™ SYBR™ Green Master Mix (Thermo Fisher Scientific) along with specific quantitative primers. The quantitative PCR primers were presented as follows: LINC00152 F: 5ʹ-GAAGGTGTCGGCAAGATC-3ʹ; R: 5ʹ-TCGGTGTCTGTCATATTCG-3ʹ. β-actin: F: 5ʹ-AAAGACCT-GTACGCCAACAC-3ʹ; R: 5ʹ-GTCATACTCCTGCTTGCTGAT-3ʹ.

### 2.4 Cell culture and grouping

The Eca 109 and Kyse 150 cells, two kinds of esophageal cancer cell lines purchased from ATCC (USA), were cultured by RPMI1640 medium (Sigma, USA) contained with 10% fetal bovine serum (FBS) (Sigma, USA) in incubator (37°C, 5% CO_2_). When the cells reached 90% fluent, 0.25% trypsin was added to digest the cells and centrifuged to discard the supernatant. The suspension were added to cell culture medium and the incubated into the cell flask and future culture. Respectively dividing the Eca 109 and Kyse 150 cells into 3 groups: Normal control (NC) groups which were treated with non-treatment; Blank (BL) groups which were transfected with empty vector (GenScript (Nanjing) Co., Ltd., China) and si-lncRNA groups which were transfected with si-lncRNA LINC00152 (GenScript (Nanjing) Co., Ltd., China).

### 2.5 MTT assay

The Eca 109 or Kyse 150 cells of different groups were collected, the cell concentration of different groups were adjusted as 1×10^5^ cells/ml, the cells were inoculated in the 96-well plate, 200 μl cell suspension were added into every holes, placed into incubator at 37°C for 48 h, 20 μl of MTT solution (5mg/ml) was added into every well, cultured in the incubator at 37℃ for 4 h, after discarding the supernatant, adding the 150 μl DMSO solution, Oscillatory reaction for 10 min, The absorbance (A) value were measured at 490 nm. The cell proliferation rates were measured.

### 2.6 The cell apoptosis rate of different groups by flow cytometry

The Eca 109 or Kyse 150 cells of different groups which were treated by different methods for 48 h were collected and adjusted as 1×10^5^ cell/ml. Taking 1 ml cell suspension, added Annexin-V buffer solution, fully mixed, added the 5 μl Propidium iodide (PI) and Annexin-V-FITC, incubated avoid light for 20min, the cell apoptosis rate of different groups were measured by flow cytometry, the experiments were repeated for 3 times.

### 2.7 Cell cycle measurement by flow cytometry

The Eca 109 or Kyse 150 cells of different groups which were treated by different methods for 48 h were collected and adjustment as 1×10^5^ cell/ml. The cells were washed by pre-cool PBS twice, the pre-cool 70% ethanol was added to fix at 4℃ overnight, washed by PBS once. After this, the centrifugation collection fixed overnight, 500 μl PBS was added to suspension cell, adding 20 μl RNase A solution and 0.2% Triton X-100. It was then cultured in a 37℃ water-bath for 30 min; added 400 μl PI Staining solution, and mixed, Incubation under light conditions at 4℃ for 30 min. The cell cycle were measured at 488 nm by Flow Cytometry. The experiments were repeated for 3 times.

### 2.8 Western blot (WB) assay

The Eca 109 or Kyse 150 cells of different groups which were logarithmic growth phase was collection. 3 ml Trizol cell lysate was added into the cells, incubated on ice for 10 min, transfer to the centrifuge tube, after centrifugation, the supernatant was transferred into the EP tube. Detecting protein concentration according to the BCA protein concentration kit. Mixed the protein with sample buffer and boiled for 5 min at 100°C, added into SDS-PAGE electrophoresis gel, 50 μl/well. After electrophoresis, proteins were transferred to PVDF (Millipore, Bedford, MA, USA) membrane at 4°C. After blocking, the membrane was incubated with primary antibodies: EGFR (1:800) (Abcam, USA), PI3K (1:800) (Abcam, USA), AKT (1:800) (Abcam, USA), P21 (1:800) (Abcam, USA) and GAPDH (1:1000) (Abcam, USA). Followed by incubation with secondary antibody (1:1000) (Abcam, USA). EGFR, PI3K, AKT and P21 gray value of protein bands were measured by Ge-1 Pro Analyzer (ver 3.0) software.

### 2.9 Statistical analysis

The data of this study were analyzed by SPSS 19.0 software, the data were expressed as Mean±SD (standard deviation). T-test was used to compare the differences between groups. The one-way ANOVA was used to analyze the differences between groups. The LSD-t test was used for multiple comparisons between groups, the value was bilateral probability, and the test level was α=0.05. *P*<0.05 indicated that the difference was statistically significant.

Ethical approval: The research related to human use has been complied with all the relevant national regulations, institutional policies and in accordance the tenets of the Helsinki Declaration, and has been approved by the authors' institutional review board or equivalent committee.

## 3 Results

### 3.1 LINC00152 expression in different tissues

**Figure 1:**
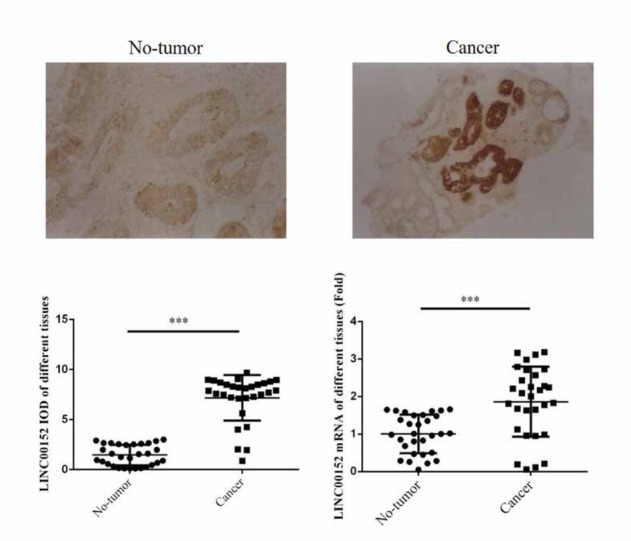
The LINC00152 expression of different tissues by ISH and RT-qPCR (×100) ***:P＜0.001, compared with no-tumor tissues

Compared with no-tumor tissues, the LINC00152 expression was significantly increased in esophageal cancer tissues (P＜0.001). By RT-qPCR, the result was shown that LINC00152 mRNA expression of esophageal cancer tissues were significantly up-regulated compared with that of non-tumor tissues. Depending on these results, we inferred that LINC00152 might be an oncology factor in esophageal cancer occur and development. The relative data was shown in Figure 1.

### 3.2 LINC 00152 affects cell proliferation rate

**Figure 2:**
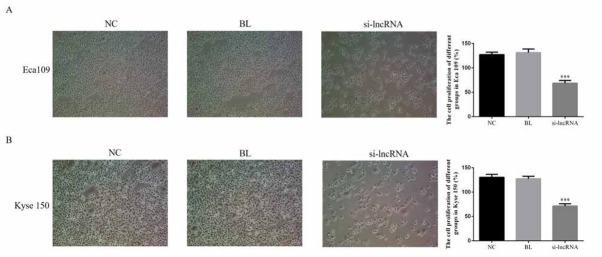
The cell proliferation rate of difference groups in Eca 109 and Kyse 150 cells. A. The Eca 109 cell proliferation rate of difference groups by MTT assay. NC: Normal control group; BL: Blank group; si-lncRNA: si-lncRNA LINC00152 group ***: P<0.05, compared with NC group. B. The Kyse 150 cell proliferation rate of difference groups by MTT assay. ***: P<0.05, compared with NC group.

To investigate the effect of the lncRNA LINC00152 on the esophageal cancer proliferation, the cells proliferation rates we measured by MTT assay. The relative results found that the lncRNA LINC00152 knockdown (si-lncRNA groups) suppressed cells proliferation compared with that of NC groups in Eca 109 (P<0.05,69.69±5.65 vs.127.33±5.02, Figure 2A) and Kyse 150 (P<0.05,71.50±4.48 vs.130.58±6.00, Figure 2B). The relative data were shown in Figure 2.

### 3.3 The cell apoptosis rates of different groups

**Figure 3:**
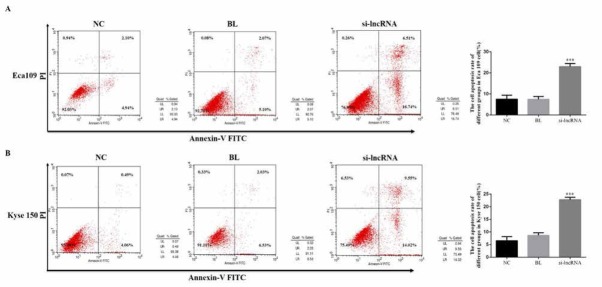
Cell apoptosis measurement by flow cytometry in Eca 109 and Kyse 150 cells. NC: Normal control group; BL: Blank group; si-lncRNA: si-lncRNA LINC00152 group A. The Eca 109 cell apoptosis rate of difference groups by flow cytometry. ***: P<0.05, compared with NC group. B. The Kyse 150 cell apoptosis rate of difference groups by flow cytometry. ***: P<0.05, compared with NC group.

To evaluate the effects of lncRNA LINC00152 in esophageal cancer apoptosis, we measured the cell apoptosis rates of difference groups by flow cytometry. Compared with NC groups, the cell apoptosis rates of si-lncRNA groups, which were knocked-down the lncRNA LINC00152, were significantly enhanced in Eca 109 (P<0.05, Figure 3A) and Kyse 150 (P<0.05, Figure 3B). The relative data were shown in Figure 3.

### 3.4 The cell cycle by flow cytometry

**Figure 4:**
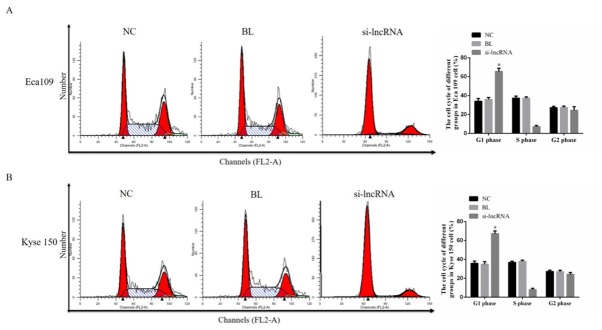
The cell cycle rates of difference groups in Eca 109 and Kyse 150 cells. NC: Normal control group; BL: Blank group; si-lncRNA: si-lncRNA LINC00152 group A. The Eca 109 cell cycle rates of difference groups by flow cytometry. ***: P<0.05, compared with NC group. B. The Kyse 150 cell cycle rates of difference groups by flow cytometry. ***: P<0.05, compared with NC group.

To investigate the effects of lncRNA LINC00152 in esophageal cancer cell cycle, the cell cycle were evaluated by flow cytometry in different groups. Compared with NC groups, the G1 phase rate of si-lncRNA groups which the lncRNA LINC00152 were down-regulated were significantly increased in Eca 109 (P<0.05, Figure 4A) and Kyse 150 (P<0.05, Figure 4B). The relative data were shown in Figure 4.

### 3.5 LINC00152 affects relative protein expression

**Figure 5:**
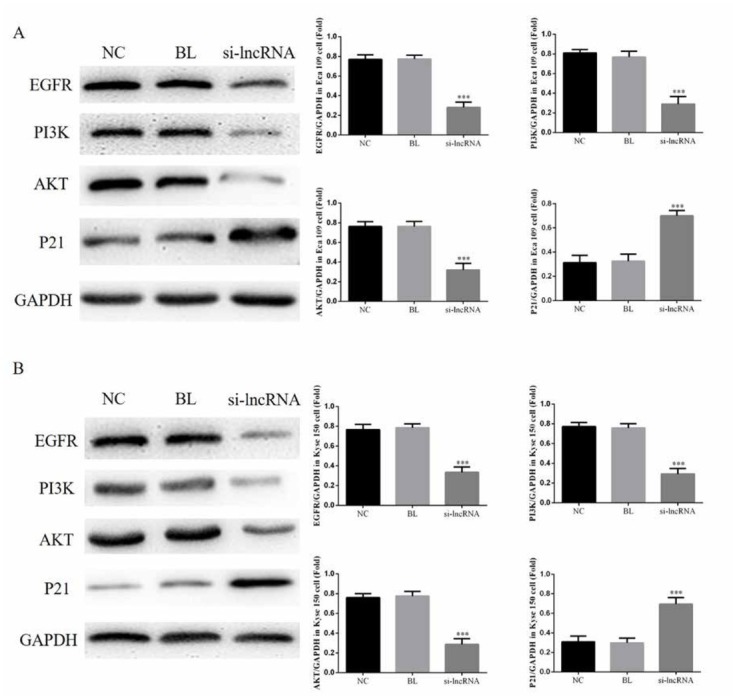
The relative proteins expressions of difference groups in Eca 109 and Kyse 150 cells. NC: Normal control group; BL: Blank group; si-lncRNA: si-lncRNA LINC00152 group A. The relative proteins expressions of difference groups in Eca 109 by WB assay. ***: P<0.05, compared with NC group. B. The relative proteins expressions of difference groups in Kyse 150 by WB assay. ***: P<0.05, compared with NC group.

The EGFR, PI3K and AKT proteins expressions of si-ln-cRNA groups were significantly suppressed compare with those proteins expression of NC groups in Eca 109 (P<0.05, Figure 5A) and Kyse 150 (P<0.05, Figure 5B). Meanwhile, the P21 protein expression of si-lncRNA group was significantly increased compared with that of NC groups in Eca 109 (P<0.05, Figure 5A) and Kyse 150 (P<0.05, Figure 5B).

## 4 Discussion

As an important role of cancer occurrence and development, lncRNAs were considered as potentially prime targets to cancer therapy. The lncRNAs are not only biomarkers, but also to be included in the prognosis of patients with various cancers [6, 7, 8, 9, 10]. Many previous studies reported that lncRNA LINC00152 were up-regulated in the cancer tissues including hepatocellular carcinoma, gastric cancer and clear cell renal carcinoma and closely related with cancer progression [11, 12, 13, 14, 15, 16]. In our present study, the results shown that knock-down of lncRNA LINC00152 suppressed esophageal cancer cell proliferation and enhanced esophageal cancer cell apoptosis. Furthermore, we analyzed the related proteins expressions with lncRNA LINC00152 down-regulation. The results showed that the expression of EGFR, PI3K and AKT proteins are inhibited, and the expression of p21 protein was stimulated by the down-expression of lncrna linc0152 in vitro study.

EGFR is a transmembrane protein on the cell surface, which is activated by homologous or heterogeneous forms. It promotes cell proliferation, differentiation and development by activating intracellular signaling pathways such as PI3K-AKT, MEK/ERK and STAT3 [17, 18, 19, 20]. PI3K/AKT pathway is a key signaling pathway in the body, and participates in the process of tumor development through regulating cell proliferation and apoptosis [21, 22, 23]. PI3K/AKT pathway also suppressed P21 expression to improve cancer cell proliferation and inhibit cell apoptosis [24]. P21 protein is the most widely known cyclin kinase inhibitor [25]. Relative studies found that P21 over-expression could keep the cell cycle in the G1 phase that might lead cell apoptosis increasing [26, 27]. In our present study, the results were shown that lncRNA LINC00152 knock-down could inhibit EGFR/PI3K/AKT pathway and stimulate P21 expression in esophageal cancer cell lines (Eca 109 and Kyse 150) in vitro study. That might be the mechanism of lncRNA LINC00152.

In conclusion, lncRNA LINC00152 knock-down might suppress esophageal cancer cell lines Eca 109 and Kyse 150 cell proliferation and induce cell apoptosis. The mechanism of lncRNA LINC00152 might be collected with EGFR/PI3K/AKT and P21 in vitro study. Depending on these results, we inferred that lncRNA LINC00152 down-regulation have anti-tumor effects via inhibiting EGFR/PI3K/AKT pathway and enhancing P21 expressions in esophageal cancer.
